# Diethyl 1,4-bis­(4-nitro­phen­yl)-1,4-dihydro-1,2,4,5-tetra­zine-3,6-dicarboxyl­ate

**DOI:** 10.1107/S1600536810002217

**Published:** 2010-01-23

**Authors:** Abdesselam Baouid, Aicha Boudina, El Hassane Soumhi

**Affiliations:** a Equipe de Chimie des Hétérocycles et Valorisation des Extraits des Plantes, Faculté des Sciences-Semlalia, Université Cadi Ayyad, Bd. Abdelkrim Khattabi, BP. 2390,40001, Marrakech, Morocco; b Equipe de Chimie des Matériaux et de l’Environnement, FSTG-Marrakech, Université Cadi Ayyad, Bd. Abdelkrim Khattabi, BP. 549, Marrakech, Morocco

## Abstract

The complete mol­ecule of the title compound, C_20_H_18_N_6_O_8_, is generated by a crystallographic twofold axis. The dihedral angle between the nitrobenzene rings is 43.5 (2)°. The central six-membered ring exhibits a boat conformation. In the crystal structure, weak inter­molecular C—H⋯O inter­actions are observed.

## Related literature

For related literature on diazepine and triazepine derivatives, see: Barltrop *et al.* (1959 ▶); Boudina *et al.* (2006 ▶); El Hazazi *et al.* (2003 ▶); Huisgen & Koch (1955 ▶); Nabih *et al.* (2003 ▶); Sharp & Hamilton (1946 ▶). For related structures, see: Chiaroni *et al.* (1995 ▶); El Hazazi *et al.* (2000 ▶).
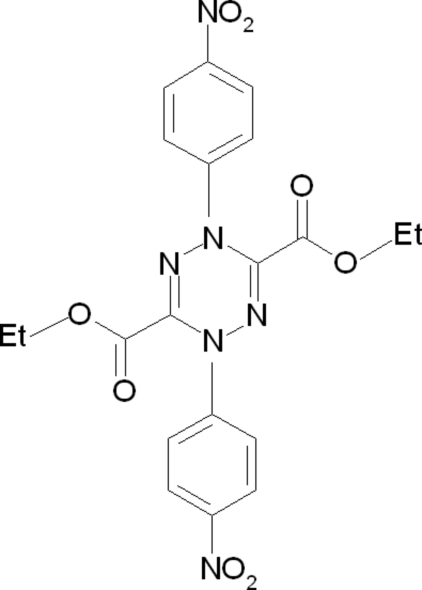


## Experimental

### 

#### Crystal data

C_20_H_18_N_6_O_8_
*M* *_r_* = 470.40
Monoclinic, 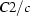

*a* = 20.739 (4) Å
*b* = 7.487 (2) Å
*c* = 14.587 (3) Å
β = 104.00 (2)°
*V* = 2197.7 (9) Å^3^
*Z* = 4
Mo *K*α radiation
μ = 0.11 mm^−1^
*T* = 300 K
0.30 × 0.15 × 0.10 mm

#### Data collection

Enraf–Nonius CAD-4 diffractometer
3158 measured reflections
2389 independent reflections
1352 reflections with *I* > 2σ(*I*)
*R*_int_ = 0.022
2 standard reflections every 60 min  intensity decay: 1.0%

#### Refinement

*R*[*F* ^2^ > 2σ(*F* ^2^)] = 0.053
*wR*(*F* ^2^) = 0.173
*S* = 1.04
2389 reflections
155 parameters
All H-atom parameters refined
Δρ_max_ = 0.27 e Å^−3^
Δρ_min_ = −0.26 e Å^−3^

### 

Data collection: *CAD-4 EXPRESS* (Enraf–Nonius, 1989 ▶); cell refinement: *CAD-4 EXPRESS*; data reduction: *MolEN* (Fair, 1990 ▶); program(s) used to solve structure: *SHELXS97* (Sheldrick, 2008 ▶); program(s) used to refine structure: *SHELXL97* (Sheldrick, 2008 ▶); molecular graphics: *ORTEPII* (Johnson, 1976 ▶); software used to prepare material for publication: *SHELXL97*.

## Supplementary Material

Crystal structure: contains datablocks I, global. DOI: 10.1107/S1600536810002217/is2514sup1.cif

Structure factors: contains datablocks I. DOI: 10.1107/S1600536810002217/is2514Isup2.hkl

Additional supplementary materials:  crystallographic information; 3D view; checkCIF report

## Figures and Tables

**Table 1:** Hydrogen-bond geometry (Å, °)

*D*—H⋯*A*	*D*—H	H⋯*A*	*D*⋯*A*	*D*—H⋯*A*
C6—H4⋯O2^i^	0.93	2.57	3.400 (4)	149
C9—H6⋯O1^ii^	0.97	2.60	3.294 (4)	129

Symmetry codes: (i) 
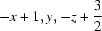
; (ii) 
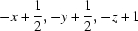
.

**Fig. 1.**
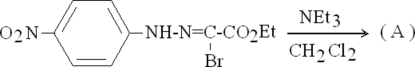
The reaction scheme of the title compound.

**Fig. 2.**
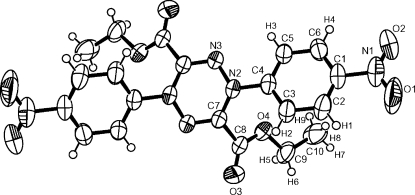
The molecule structure of the title compound with 50% probability ellipsoids.

## supplementary crystallographic information

### Comment

In order to prepare a new heterocyclic systems, our research team have been interested in the 1,3-dipolar cycloaddition reaction of nitrile oxides and nitrilimines toward diazepines, benzodiazepines, triazepines and benzotriazepines (El Hazazi *et al.*, 2003; Nabih *et al.*, 2003; Boudina *et al.*, 2006). In this context, we directed our axe of research to examine reactivity of adducts-obtained from the 1,5-benzodiazepine (Barltrop *et al.*, 1959) *via* 1,3-dipolar cycloaddition reaction of nitrilimines- with *N*-paranitrophenylnitrilimine (Sharp & Hamilton, 1946; Huisgen & Koch, 1955). This reaction provided to bicycloadduct and a new heterocycle (A). The new heterocycle (A) resulted from precursor ethyl paranitrophenylhydrazono-α-bromoglyoxylate by the action of triethylamine in dichloromethane at room temperature (Fig. 1).

The structure of product (A) was determined on the basis of NMR spectral data (1H and 13 C) and studied by single-crystal X-ray diffraction (Fig. 2). The asymmetric unit consists of one independent [C_10_H_9_N_3_O_4_] group that form one half of a molecule compound. The main geometric features of this group are in good agreement with those observed in similar compounds (Chiaroni *et al.*, 1995; El Hazazi *et al.*, 2000). The molecule of nominal compound is localized around a twofold rotation axis and exhibit a boat conformation in which N2 and N2a show the maximum deviation (0.3213 Å) from N3/N2/C7/N3^i^/N2^i^/C7^i^ plane [symmetry code: (i) -*x* + 1, *y*, -*z* + 1/2].

### Experimental

Triethylamine (0.8 mmol) in dichloromethane (2 ml) was added at room temperature to a stirred solution of ethyl paranitrophenylhydrazono-α-bromoglyoxylate (0.65 mmol) in dichloromethane (20 ml). The mixture was stirred at room temperature, washed with water and the aqueous phase was then extracted with ether (3×20 ml). The organic layer was dried over anhydrous sodium sulfate and then concentrated under reduced pressure and recrystallized from ethanol to afford the reaction product (A) (m.p. = 219–220 °C).

### Refinement

All H atoms were located in a difference map and refined using a riding model, with d(C—H) = 0.93–0.97 Å, and with *U*_iso_(H) =1.2*U*_eq_(C).

### Crystal data

**Table:**

C_20_H_18_N_6_O_8_	*F*(000) = 976
*M**_r_* = 470.40	*D*_x_ = 1.422 Mg m^−^^3^
Monoclinic, *C*2/*c*	Mo *K*α radiation, λ = 0.71073 Å
Hall symbol: -C 2yc	Cell parameters from 25 reflections
*a* = 20.739 (4) Å	θ = 10–15°
*b* = 7.487 (2) Å	µ = 0.11 mm^−^^1^
*c* = 14.587 (3) Å	*T* = 300 K
β = 104.00 (2)°	Prism, colourless
*V* = 2197.7 (9) Å^3^	0.30 × 0.15 × 0.10 mm
*Z* = 4	

### Data collection

**Table:**

Enraf–Nonius CAD-4 diffractometer	*R*_int_ = 0.022
Radiation source: fine-focus sealed tube	θ_max_ = 27.0°, θ_min_ = 2.0°
graphite	*h* = −26→25
ω/2θ scans	*k* = −9→2
3158 measured reflections	*l* = −1→18
2389 independent reflections	2 standard reflections every 60 min
1352 reflections with *I* > 2σ(*I*)	intensity decay: 1.0%

### Refinement

**Table:**

Refinement on *F*^2^	Primary atom site location: structure-invariant direct methods
Least-squares matrix: full	Secondary atom site location: difference Fourier map
*R*[*F*^2^ > 2σ(*F*^2^)] = 0.053	Hydrogen site location: difference Fourier map
*wR*(*F*^2^) = 0.173	All H-atom parameters refined
*S* = 1.04	*w* = 1/[σ^2^(*F*_o_^2^) + (0.080*P*)^2^ + 0.9509*P*] where *P* = (*F*_o_^2^ + 2*F*_c_^2^)/3
2389 reflections	(Δ/σ)_max_ < 0.001
155 parameters	Δρ_max_ = 0.27 e Å^−^^3^
0 restraints	Δρ_min_ = −0.26 e Å^−^^3^

### Special details

**Table:**

Geometry. All e.s.d.'s (except the e.s.d. in the dihedral angle between two l.s. planes) are estimated using the full covariance matrix. The cell e.s.d.'s are taken into account individually in the estimation of e.s.d.'s in distances, angles and torsion angles; correlations between e.s.d.'s in cell parameters are only used when they are defined by crystal symmetry. An approximate (isotropic) treatment of cell e.s.d.'s is used for estimating e.s.d.'s involving l.s. planes.
Refinement. Refinement of *F*^2^ against ALL reflections. The weighted *R*-factor *wR* and goodness of fit *S* are based on *F*^2^, conventional *R*-factors *R* are based on *F*, with *F* set to zero for negative *F*^2^. The threshold expression of *F*^2^ > σ(*F*^2^) is used only for calculating *R*-factors(gt) *etc*. and is not relevant to the choice of reflections for refinement. *R*-factors based on *F*^2^ are statistically about twice as large as those based on *F*, and *R*- factors based on ALL data will be even larger.

### Fractional atomic coordinates and isotropic or equivalent isotropic displacement parameters (Å^2^)

**Table:**

	*x*	*y*	*z*	*U*_iso_*/*U*_eq_	
O1	0.32809 (17)	0.4483 (5)	0.63327 (19)	0.1695 (16)	
O2	0.41693 (14)	0.3584 (4)	0.72174 (17)	0.1199 (10)	
O3	0.32302 (9)	0.0896 (4)	0.16616 (14)	0.1091 (9)	
O4	0.37172 (7)	−0.0126 (3)	0.31106 (12)	0.0661 (5)	
N1	0.38276 (16)	0.3893 (3)	0.6442 (2)	0.0884 (8)	
N2	0.47726 (9)	0.2300 (3)	0.32241 (12)	0.0540 (5)	
N3	0.54350 (8)	0.1630 (3)	0.33902 (12)	0.0564 (5)	
C1	0.40750 (14)	0.3493 (3)	0.55998 (17)	0.0636 (7)	
C2	0.36884 (14)	0.3910 (4)	0.4717 (2)	0.0716 (8)	
H1	0.3273	0.4430	0.4653	0.086*	
C3	0.39257 (13)	0.3545 (3)	0.39242 (18)	0.0654 (7)	
H2	0.3678	0.3856	0.3325	0.078*	
C4	0.45413 (11)	0.2706 (3)	0.40419 (15)	0.0520 (5)	
C5	0.49295 (12)	0.2332 (3)	0.49362 (16)	0.0590 (6)	
H3	0.5346	0.1811	0.5008	0.071*	
C6	0.46928 (13)	0.2741 (4)	0.57236 (17)	0.0660 (7)	
H4	0.4950	0.2507	0.6328	0.079*	
C7	0.43585 (10)	0.1682 (3)	0.23753 (15)	0.0541 (6)	
C8	0.36944 (12)	0.0780 (4)	0.23316 (18)	0.0651 (7)	
C9	0.30977 (13)	−0.0998 (5)	0.3205 (2)	0.0867 (10)	
H5	0.3033	−0.2111	0.2854	0.104*	
H6	0.2720	−0.0227	0.2954	0.104*	
C10	0.31545 (15)	−0.1344 (5)	0.4218 (2)	0.1016 (12)	
H7	0.2749	−0.1864	0.4299	0.122*	
H8	0.3236	−0.0241	0.4562	0.122*	
H9	0.3516	−0.2153	0.4453	0.122*	

### Atomic displacement parameters (Å^2^)

**Table:**

	*U*^11^	*U*^22^	*U*^33^	*U*^12^	*U*^13^	*U*^23^
O1	0.175 (3)	0.249 (4)	0.118 (2)	0.124 (3)	0.100 (2)	0.043 (2)
O2	0.144 (2)	0.165 (3)	0.0653 (13)	0.0037 (18)	0.0534 (14)	−0.0131 (15)
O3	0.0536 (11)	0.199 (3)	0.0711 (12)	−0.0147 (14)	0.0087 (10)	0.0158 (15)
O4	0.0476 (9)	0.0843 (12)	0.0706 (10)	−0.0101 (8)	0.0223 (7)	0.0018 (9)
N1	0.119 (2)	0.0823 (17)	0.0852 (18)	0.0141 (15)	0.0671 (17)	0.0024 (14)
N2	0.0471 (10)	0.0709 (13)	0.0503 (10)	0.0008 (9)	0.0237 (8)	−0.0022 (9)
N3	0.0449 (10)	0.0765 (14)	0.0516 (11)	−0.0025 (9)	0.0187 (8)	−0.0018 (9)
C1	0.0872 (18)	0.0555 (14)	0.0619 (14)	0.0005 (13)	0.0450 (13)	−0.0020 (12)
C2	0.0814 (17)	0.0651 (16)	0.0833 (18)	0.0191 (13)	0.0493 (15)	0.0105 (14)
C3	0.0725 (15)	0.0698 (17)	0.0634 (14)	0.0164 (13)	0.0354 (12)	0.0112 (12)
C4	0.0581 (13)	0.0524 (13)	0.0528 (12)	−0.0049 (10)	0.0276 (10)	−0.0017 (10)
C5	0.0563 (13)	0.0717 (16)	0.0537 (13)	−0.0029 (12)	0.0225 (10)	−0.0057 (12)
C6	0.0762 (17)	0.0738 (17)	0.0533 (13)	−0.0050 (14)	0.0258 (12)	−0.0059 (12)
C7	0.0455 (11)	0.0700 (15)	0.0510 (12)	0.0068 (11)	0.0200 (10)	0.0026 (11)
C8	0.0433 (12)	0.099 (2)	0.0579 (14)	0.0004 (12)	0.0217 (11)	−0.0058 (14)
C9	0.0569 (15)	0.121 (3)	0.092 (2)	−0.0301 (16)	0.0365 (14)	−0.0204 (18)
C10	0.0654 (17)	0.130 (3)	0.110 (2)	−0.0187 (18)	0.0216 (17)	0.046 (2)

### Geometric parameters (Å, °)

**Table:**

O1—N1	1.191 (3)	C3—C4	1.396 (3)
O2—N1	1.202 (3)	C3—H2	0.9300
O3—C8	1.198 (3)	C4—C5	1.386 (3)
O4—C8	1.314 (3)	C5—C6	1.388 (3)
O4—C9	1.477 (3)	C5—H3	0.9300
N1—C1	1.473 (3)	C6—H4	0.9300
N2—C7	1.403 (3)	C7—N3^i^	1.290 (3)
N2—C4	1.422 (3)	C7—C8	1.522 (3)
N2—N3	1.427 (3)	C9—C10	1.478 (4)
N3—C7^i^	1.290 (3)	C9—H5	0.9700
C1—C6	1.371 (4)	C9—H6	0.9700
C1—C2	1.378 (4)	C10—H7	0.9600
C2—C3	1.389 (3)	C10—H8	0.9600
C2—H1	0.9300	C10—H9	0.9600
			
C8—O4—C9	117.3 (2)	C6—C5—H3	120.2
O1—N1—O2	121.5 (3)	C1—C6—C5	119.2 (2)
O1—N1—C1	118.4 (3)	C1—C6—H4	120.4
O2—N1—C1	120.0 (3)	C5—C6—H4	120.4
C7—N2—C4	123.50 (18)	N3^i^—C7—N2	120.89 (19)
C7—N2—N3	113.10 (17)	N3^i^—C7—C8	115.9 (2)
C4—N2—N3	116.03 (18)	N2—C7—C8	122.52 (19)
C7^i^—N3—N2	110.49 (18)	O3—C8—O4	126.6 (2)
C6—C1—C2	122.0 (2)	O3—C8—C7	122.9 (2)
C6—C1—N1	118.5 (2)	O4—C8—C7	110.5 (2)
C2—C1—N1	119.5 (2)	O4—C9—C10	108.1 (2)
C1—C2—C3	119.4 (2)	O4—C9—H5	110.1
C1—C2—H1	120.3	C10—C9—H5	110.1
C3—C2—H1	120.3	O4—C9—H6	110.1
C2—C3—C4	118.9 (2)	C10—C9—H6	110.1
C2—C3—H2	120.5	H5—C9—H6	108.4
C4—C3—H2	120.5	C9—C10—H7	109.5
C5—C4—C3	120.8 (2)	C9—C10—H8	109.5
C5—C4—N2	120.6 (2)	H7—C10—H8	109.5
C3—C4—N2	118.5 (2)	C9—C10—H9	109.5
C4—C5—C6	119.6 (2)	H7—C10—H9	109.5
C4—C5—H3	120.2	H8—C10—H9	109.5
			
C7—N2—N3—C7^i^	42.9 (2)	N2—C4—C5—C6	180.0 (2)
C4—N2—N3—C7^i^	−165.9 (2)	C2—C1—C6—C5	−2.1 (4)
O1—N1—C1—C6	−177.2 (3)	N1—C1—C6—C5	179.1 (2)
O2—N1—C1—C6	1.1 (4)	C4—C5—C6—C1	0.6 (4)
O1—N1—C1—C2	3.9 (4)	C4—N2—C7—N3^i^	167.8 (2)
O2—N1—C1—C2	−177.7 (3)	N3—N2—C7—N3^i^	−43.5 (3)
C6—C1—C2—C3	0.6 (4)	C4—N2—C7—C8	−21.9 (4)
N1—C1—C2—C3	179.4 (2)	N3—N2—C7—C8	126.8 (2)
C1—C2—C3—C4	2.3 (4)	C9—O4—C8—O3	−4.4 (4)
C2—C3—C4—C5	−3.7 (4)	C9—O4—C8—C7	176.5 (2)
C2—C3—C4—N2	178.5 (2)	N3^i^—C7—C8—O3	−40.8 (4)
C7—N2—C4—C5	143.2 (2)	N2—C7—C8—O3	148.5 (3)
N3—N2—C4—C5	−4.6 (3)	N3^i^—C7—C8—O4	138.4 (2)
C7—N2—C4—C3	−39.0 (3)	N2—C7—C8—O4	−32.3 (3)
N3—N2—C4—C3	173.1 (2)	C8—O4—C9—C10	−159.4 (3)
C3—C4—C5—C6	2.3 (4)		

Symmetry codes: (i) −*x*+1, *y*, −*z*+1/2.

### Hydrogen-bond geometry (Å, °)

**Table:**

*D*—H···*A*	*D*—H	H···*A*	*D*···*A*	*D*—H···*A*
C6—H4···O2^ii^	0.93	2.57	3.400 (4)	149
C9—H6···O1^iii^	0.97	2.60	3.294 (4)	129

Symmetry codes: (ii) −*x*+1, *y*, −*z*+3/2; (iii) −*x*+1/2, −*y*+1/2, −*z*+1.
